# Development and content validity of a website for patients with coronary artery disease

**DOI:** 10.1590/0034-7167-2022-0302

**Published:** 2023-01-30

**Authors:** Luiz Felipe Gonçalves Arroio, Juliana de Lima Lopes, Alba Lúcia Bottura Leite de Barros, Edvone Alves de Lima, Camila Tákao Lopes, Vinicius Batista Santos

**Affiliations:** I Universidade Federal de São Paulo. São Paulo, São Paulo, Brazil

**Keywords:** Computer Communication Networks, Coronary Artery Disease, Secondary Prevention, Health Education, Validation Study., Redes de Comunicación de Computadores, Enfermedad de la Arteria Coronaria, Prevención Secundaria, Educación en Salud, Estudio de Validación, Redes de Comunicação de Computadores, Doença da Artéria Coronariana, Prevenção Secundária, Educação em Saúde, Estudos de Validação.

## Abstract

**Objectives:**

to develop and analyze content validity evidence of a website for patients with coronary artery disease.

**Methods:**

a methodological study, carried out in the phases: Definition - determined contents for inclusion in the website, architecture and design; Implementation - subjects included in the website; Assessment - website submitted to analysis by 13 experts and eight laypersons regarding organization, content and design, on a scale of 1 (no agreement) to 4 (complete agreement). Items that reached Content Validity Ratio (CVR) higher than the established critical values and Content Validity Index greater than 0.80 were considered valid.

**Results:**

eight domains related to secondary prevention in coronary heart disease were included on the website. Critical CVR and adequate CVI were obtained according to professional and lay experts.

**Conclusions:**

the website was developed, achieving adequate content validity evidence, and can be used as an educational tool for this population.

## RESUMO

**Objetivos:**

desenvolver e analisar as evidências de validade de conteúdo de um *website* para pacientes com doença arterial coronariana.

**Métodos:**

estudo metodológico, realizado nas fases: Definição - determinado os conteúdos para inclusão no *website*, arquitetura e *design*; Implementação - assuntos incluídos no *website*; Avaliação - *website* submetido à análise por 13 especialistas e oito leigos quanto à organização, conteúdo e *design*, em uma escala de 1 (não concordância) a 4 (total concordância). Considerados válidos os itens que atingiram uma Razão de Validade de Conteúdo (CVR) superior aos valores críticos estabelecidos e um Índice de Validade de Conteúdo superior a 0,80.

**Resultados:**

incluídos no *website* oito domínios relacionados à prevenção secundária em coronariopatias. Um CVR crítico e um IVC adequado foram obtidos na opinião de especialistas profissionais e leigos.

**Conclusões:**

o *website* foi desenvolvido, alcançando adequadas evidências de validade de conteúdo, e pode ser utilizado como ferramenta educacional para esta população.

## RESUMEN

**Objetivos:**

desarrollar y analizar evidencias de validez de contenido de un sitio web para pacientes con enfermedad arterial coronaria.

**Métodos:**

estudio metodológico, realizado en las siguientes fases: Definición - determinado los contenidos para su inclusión en el sitio web, la arquitectura y el diseño; Implementación - temas incluidos en el sitio web; Evaluación - sitio web sometido a análisis por 13 expertos y ocho legos en cuanto a organización, contenido y diseño, en una escala de 1 (sin acuerdo) a 4 (totalmente de acuerdo). Se consideran válidos los artículos que alcanzaron un Índice de Validez de Contenido (CVR) superior a los valores críticos establecidos y un Índice de Validez de Contenido superior a 0,80.

**Resultados:**

se incluyeron en el sitio web ocho dominios relacionados con la prevención secundaria en enfermedades coronarias. Se obtuvo un CVR crítico y un CVI adecuado en opinión de expertos profesionales y legos.

**Conclusiones:**

se elaboró el sitio web, lográndose evidencias adecuadas de validez de contenido, pudiendo ser utilizado como herramienta educativa para esta población.

## INTRODUCTION

Cardiovascular diseases (CVD) are the leading causes of death in the world. According to data for the year 2021, it is estimated that more than 19 million people died from CVD, which represents an increase of 18.7% when compared to 2010 data. Of these deaths, it is estimated that 7.4 million occur due to ischemic heart diseases, and 6.7 million due to strokes^(1)^.

Coronary artery disease (CAD) is the result of obstruction of coronary arteries by fatty plaque deposits, usually secondary to exposure to cardiovascular risk factors. Risk factors may be related to lifestyle, such as a sedentary lifestyle, inadequate diet, tobacco use and harmful use of alcohol, as well as physiological changes, such as systemic arterial hypertension, diabetes mellitus and dyslipidemia. There are also non-modifiable risk factors, such as family history, age, sex, and race^(2-4)^.

To prevent the progression and establishment of atherosclerosis, it is necessary to have cardiovascular prevention programs that focus on lifestyle modification through the adoption of healthy practices, such as smoking cessation, reducing salt in the diet, increasing the consumption of fruits, vegetables and vegetables, increasing the level of regular physical activity and reducing harmful use of alcohol, in addition to helping patients to adhere to drug for comorbidities^(4-5)^.

Cardiovascular prevention can be categorized as primary and secondary, the primary being the set of actions that aim to reduce the disease incidence in the population, removing the causal factors. Secondary prevention, in turn, consists of the set of actions that aim to identify and correct, as early as possible, any deviation from normality, in order to immediately put the individual in a healthy situation; i.e., it aims to reduce the disease prevalence, favoring the previously diagnosed individual to adopt a healthier lifestyle, thus reducing cardiovascular risks^(3,5-6)^.

One of the strategies used in secondary prevention programs is health education, involving the use of one or several instructional methods to achieve the expected results, such as group lectures, individualized instruction, games, simulation, role-play and self-instruction modules via websites^(7)^.

Faced with technological growth and large-scale access to the internet, use of technology in health education has been gaining ground, being increasingly used by professionals in the area. Considering that 26% of Brazilians look for health information on the internet, that popular information regarding CAD is scarce and that health education represents a fundamental strategy in the process of forming behaviors that promote and maintain health^(8)^, it is relevant that professionals are committed to making available and/or disseminate valid and reliable materials online.

## OBJECTIVES

To develop and analyze content validity evidence of a website for patients with CAD.

## METHODS

### Ethical aspects

This project was submitted and approved by the teaching institution’s Research Ethics Committee (REC), and meets the scientific requirements in treatment of subjects participating in research. The judges were assured confidentiality and anonymity.

### Study design

This is a methodological study of construction and analysis of content validity evidence of a website. The theoretical framework adopted for website construction and validity was proposed by a graphic designer, Clement Mok, called DADI (definition, architecture, design, implementation and assessment), which allows a better organization for operating the website development^(9-10)^.

### Definition phase

At this stage, the contents and the way in which content was presented on the website were determined. To this end, information related to secondary prevention for patients with CAD was extracted from articles on lifestyle modification. The data sources consulted were PubMed (National Library of Medicine), CINAHL (Cumulative Index to Nursing and Allied Health Literature), VHL (Virtual Health Library), including BDENF (*Base de Dados em Enfermagem*), MEDLINE (Medical Literature Analysis and Retrieval System Online), SciELO (Scientific Electronic Library Online) and LILACS (Latin American and Caribbean Literature in Health Sciences).

The texts that guided the website’s subjects were initially extracted from national and international guidelines on cardiovascular prevention^(2-6)^. For each subject, articles published, preferably from the last 5 years, were selected that provided information regarding the management of the main cardiovascular risk factors and also guidelines on the CAD itself (symptoms, clinical and interventional treatment).

Eight main topics were selected, being called “instructional domains”. To convey information on the selected topics, it was decided to develop booklets, animated videos, interviews with professionals and banners, in order to facilitate learning and retention of content, and these materials were developed specifically for their insertion on the website.

### Architecture and design phase

In this phase, the website creation, structuring and testing was carried out. Based on the contents identified and produced in the previous phase, the website layout and type of programming were developed. The following applications were used for architecture and design: Dreamweaver CS3^®^, for page building, and Photoshop CS3 and Flash^®^ CS3, for image editing, logo creation and animation from the top of the website. At this stage, the project had the collaboration of a professional from an Information Technology institution to organize the subjects on the website. The website was hosted on the homepage: www.educacor.unifesp.br.

### Implementation phase

This phase involved publishing the website on the internet.

### Assessment phase

At this stage, the website was assessed by professional and lay experts. After the website was developed, 15 professional experts were initially invited and given a period of one month for review, of which 13 completed the assessment.

Professionals should have a *lato sensu* graduate degree in cardiology, with clinical practice of at least two years in the area. Sampling was of the snowball type, the first stratum being carried out from the search on the *Platforma Lattes* of the Brazilian National Council for Scientific and Technological Development (CNPq - *Conselho Nacional de Desenvolvimento Cientifico e Tecnológico*) portal, or by prior knowledge of the researchers, who publish in the area of cardiology and prevention secondary in cardiology.

The website was analyzed in terms of general assessment, i.e., presence of website authorship, graphic design, topic organization, user attention and link easiness. For each instructional domain, the following were assessed: clarity of information; amount of information; topic organization; information reliability; relationship of figures with the text; if figures added knowledge; and presence of grammatical errors. The experts assigned grades from 1 to 4 for each indicator, with grade 1 referring to the criterion of poor, 2, fair, 3, good, and 4, excellent^(9)^, and could leave comments and suggestions that they deemed necessary.

Sequentially, the website was assessed by lay people, recruited through snowball sampling, the first stratum being due to the authors’ prior knowledge. Initially, 10 lay people were invited, eight of whom completed the assessment. Lay people should have a previous diagnosis of CAD, be over 18 years of age, literate and routinely use computers with internet browsing. Lay people assessed the website regarding the general content (adequacy of information, organization and identification of information, quality of information, whether the link is adequate) and also regarding clarity of information, the relationship of figures with text and if figures and texts added knowledge. Assessment was performed using the same scale from 1 to 4 (1 = poor, 2 = fair, 3 = good and 4 = excellent)^(9)^.

For both professional and lay experts, the invitation to participate in the study was sent by email. After digitally signing the Informed Consent Form, the website link and a Google Forms form were sent to assess the website.

### Data analysis

Descriptive statistics were performed to characterize professional and lay experts. For content validity evidence analysis, initially, the Content Validity Ratio was calculated, using the formula^(11)^:


$$CVR=\frac{ne-(N/2)}{N/2}$$

Thus, ne - number of experts who scored 3 or 4; and N - number of judges. Considering the sample of 13 professional experts, the CVR was calculated considering the minimum value of 0.53 at a significance level of 0.04. For layperson analysis, considering the feedback of eight people, the CVR was calculated considering the minimum value of 0.75 at a significance level of 0.03^(11)^.

After calculating the CVR, the Content Validity Index (CVI) was calculated (number of experts who scored 3 or 4 divided by the number of experts/lay people), with a CVI greater than 0.80 being considered valid^(12)^.

## RESULTS

The website, called EDUCACOR, was developed with two fictional characters, called “nurse Julia” and “Hearty”, an anthropomorphized heart. The website was divided into nine instructional domains, based on the identification of the main topics related to CAD, namely: 1. website presentation; 2. coronary heart disease; 3. non-drug treatment; 4. drug treatment; 5. diet; 6. physical activity; 7. smoking and alcohol; 8. stress and sleep; 9. sexual activity.

**Figure 1:**
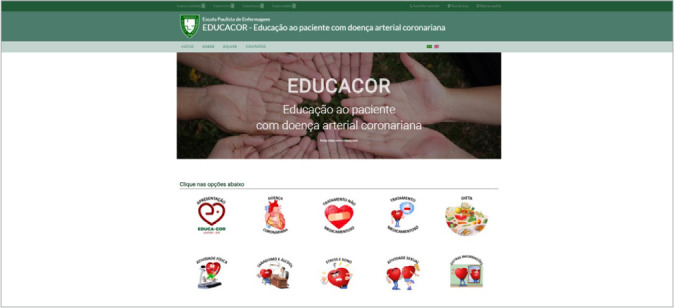
EDUCACOR website initial presentation, São Paulo, São Paulo, Brazil, 2020

The website developed has the character of being of free access, not requiring prior registration, to facilitate its access to the population of patients and family members of patients with CAD. The website initial presentation consists of a launch banner and four launch buttons, where the purpose of the website, the project team and the contact of the person in charge are described and, just below, ten icons for each instructional domain in figure format (Figure 1).

The presentation icon contains explanations about the website objectives, who are the professionals and students involved, what were the motivations for creating the website and a video of those responsible, explaining their personal motivations for its construction.

In the coronary disease domain, the pathophysiology of CAD was addressed, as well as the signs and symptoms in a simple way, so that patients understand the formation of atherosclerotic plaque and its relationship with some diseases and lifestyle, through an explanatory video of a fictitious history of a patient, being complemented with a video in which an interview was carried out by a nurse researcher in the field of cardiology.

In the non-drug treatment domain, the possible treatments that can be performed in people with CAD were addressed, focusing on percutaneous coronary intervention and coronary artery bypass graft surgery, through a booklet containing information on the types of treatments and recovery. Also, a video was added telling the story of a fictional character, who would undergo a percutaneous coronary intervention, called “Mr. João”.

With regard to the drug treatment domain, the drugs most frequently used in the treatment of patients with CAD were discussed as well as strategies so that patients do not forget to take them and the possible places to purchase the drugs.

**Figure 2:**
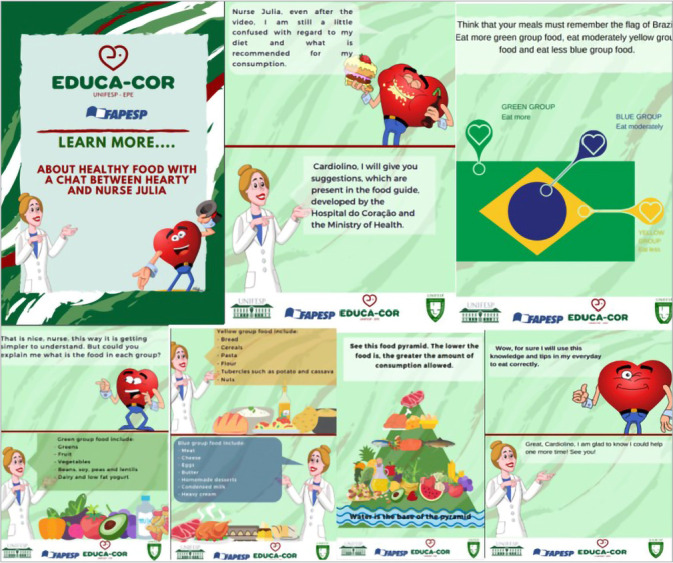
Excerpt from the booklet on healthy eating inserted in the website diet domain, São Paulo, São Paulo, Brazil, 2020

In the diet domain, guidance on cardioprotective foods indicated for this population was included through a fictional story of “Ms. Neusa” and the importance of incorporating a healthy diet into daily life, in addition to a booklet containing foods indicated for secondary prevention and a banner on tips for healthy eating (Figure 2).

Regarding the physical activity domain, a booklet was developed on the importance of physical activity in blood pressure control, glycemic and lipid levels and quality of life, in addition to a video containing guidelines on the safe practice of physical activity. The booklet was developed by a nurse with a degree in physical education, in addition to a banner containing tips for a safe walk, which included information about clothing, food and clinical signs that should be observed.

Smoking and alcohol consumption were addressed in a single domain, consisting of an explanatory video about smoking and its impact on heart health, a booklet reinforcing what has already been said in the video and adding information about treatments offered by the Unified Health System (SUS - *Sistema Único de Saúde*), for smoking and alcoholism cessation, in addition to the existing addresses and telephone numbers for the treatment of their dependence.

The topics addressed in the stress and sleep domain included the effect of stress and poor sleep quality on the lives of people with CAD, including a booklet with several possibilities for improving sleep and stress. In the sexual activity domain, guidelines on how to practice sexual activity safely after an acute coronary event were included, through a video and a booklet containing the main recommended sexual positions for men and women after acute myocardial infarction.

The website was assessed by 13 health professionals, most of whom were women, with an average age of 36 years, with 46% having a maximum degree of specialization, 39% a master’s degree and 15% a doctoral degree. Regarding the area of activity, 30% worked in assistance, 15% in research, 15% in teaching and 40% in more than one area.

Regarding the website general assessment (information of those responsible, contact of the authors, graphic design, topic organization, user attention and link easiness), all indicators obtained a CVR above the critical value established for the number of experts who assessed the website. In relation to CVI, all indicators obtained a value higher than 0.90.

Table 1 shows the value of CVR and CVI for each indicator assessed, according to the instructional domain, demonstrating that all indicators obtained a CVR equal to or greater than the recommended limit, as well as the CVI value, except in domain 3, which presented typing errors during the experts’ assessment, and in domain 7, in which the exclusion of interventions to control stress was recommended, as they are not common practices in the Brazilian reality (Yoga and Tai Chi Chuan), according to the number of responding experts.

**Table 1:** Content Validity Ratio/Content Validity Index of the indicators of each website domain according to expert assessment, São Paulo, São Paulo, Brazil, 2022

	1	2	3	4	5	6	7	8
Clarity of information	0.69 0.84	1.00 1.00	1.00 1.00	1.00 1.00	1.00 1.00	0.84 0.92	0.69 0.84	0.84 0.92
Quantity of information	1.00 1.00	0.84 0.92	1.00 1.00	1.00 1.00	0.69 0.84	0.84 0.92	0.53 0.76	0.84 0.92
Organization	0.84 0.92	1.00 1.00	1.00 1.00	0.84 0.92	0.69 0.84	1.00 1.00	084 0.92	1.00 1.00
Reliability of information	1.00 1.00	0.84 0.92	1.00 1.00	1.00 1.00	0.84 0.92	1.00 1.00	0.69 0.84	1.00 1.00
Relationship of figures with text	0.84 0.92	0.84 0.92	0.84 0.92	1.00 1.00	0.69 0.84	0.84 0.92	0.84 0.92	1.00 1.00
Figures add knowledge	0.69 0.84	0.84 0.92	1.00 1.00	1.00 1.00	0.84 0.92	0.84 0.92	1.00 1.00	0.84 0.92
Grammatical errors	1.00 1.00	1.00 1.00	0.53 0.76	0.84 0.92	1.00 1.00	0.69 0.84	1.00 1.00	0.69 0.84

*1 - coronary artery disease; 2 - non-drug treatment; 3 - drug treatment; 4 - diet; 5 - physical activity; 6 - smoking and alcohol; 7 - stress and sleep; 8 - sexual activity.*

Some textual changes were suggested in relation to the type of word for a better understanding of the lay population, modification of some colors in the booklets to avoid reading strain, insertion of examples of more healthy foods and to improve page organization in the physical activity domain.

After the changes made, the website was assessed by eight lay people, most of whom were women, with an average age of 56 years, 12.5% with graduate degrees, 50% with higher education and 37.5% with complete secondary education. In the assessment in relation to the website general aspects with regard to information about the research team members, contact with the authors, link easiness, and ease of navigation, a CVR/CVI equal to 1.0 and in the topic organization indicator, CVR of 0.75 and a CVI of 0.87.

For all indicators assessed for each instructional domain, a CVR equal to or greater than the critical value established by the number of lay respondents was obtained, as well as a CVI greater than 0.80, according to Table 2.

**Table 2:** Content Validity Ratio/Content Validity Index of the indicators of each domain of the website according to lay people’s assessment, São Paulo, São Paulo, Brazil, 2022

	1	2	3	4	5	6	7	8
Clarity of information	1.00 1.0	1.00 1.0	0.75 0.87	0.75 0.87	0.75 0.87	0.75 0.87	0.75 0.87	1.00 1.00
Relationship of figures with text	1.00 1.00	0.75 0.87	0.75 0.87	0.75 0.87	0.75 0.87	0.75 0.87	0.75 0.87	1.00 1.00
Information added knowledge	0.75 0.87	0.75 0.87	0.75 0.87	0.75 0.87	0.75 0.87	0.75 0.87	0.75 0.87	1.00 1.00
Figures added knowledge	0.75 0.87	0.75 0.87	0.75 0.87	0.75 0.87	0.75 0.87	0.75 0.87	0.75 0.87	1.00 1.00

*1 - coronary artery disease; 2 - non-drug treatment; 3 - drug treatment; 4 - diet; 5 - physical activity; 6 - smoking and alcohol; 7 - stress and sleep; 8 - sexual activity.*

## DISCUSSION

Population aging, associated with chronic diseases, has become one of the main public health issues. Considering that the objective of primary care is to bring health care as close as possible to the places where people live and work, through longitudinality, comprehensiveness and coordination of care, measures that help patients and their families in treatment compliance should be implemented. One of these measures is the use of health technology^(13)^.

When the perception of multidisciplinary teams on health education practices and on the role of nurses in the performance of educational activities are clear, patients receive a much more in-depth, direct and individualized education in the face of their desires. The acquisition of knowledge associated with multidisciplinary CAD control programs helps patients to obtain a more adequate perception of their health status, enabling the modification of beliefs, behaviors and bad habits^(13)^.

The use of educational materials, together with the guidance of multidisciplinary teams, has been widely disseminated and contributes positively to the improvement of risk factors, knowledge about the disease and compliance with the proposed treatment^(14-15)^. In this context, the EDUCACOR website was developed and validated by professional and lay experts. Each domain addressed a different subject, seeking to bring, as broadly and completely as possible, information about CAD, risk factors, preventive measures and the importance of treatment compliance.

The domains related to CAD and treatment brought information regarding the pathophysiology of CAD regarding the formation of atherosclerotic plaque and which symptoms related to the disease that patients should monitor, in addition to explanations about percutaneous coronary intervention and surgical myocardial revascularization surgery, especially in relation to procedure description and care after performing this treatment^(2-6)^. All this information was supported by the latest national and international guidelines on CAD^(2-6)^ and also by the Theory of Self-Care of Chronic Illness assumptions: self-monitoring is related to the identification of clinical signs of health problems and patients with better knowledge of the disease tend to have greater self-monitoring and control^(16)^.

The domain related to drug treatment addressed the drugs that are usually prescribed by the doctor, their indications and recommendations for the time of use. Interventions that can increase drug compliance and reduce forgetfulness were also addressed in this matter, such as drawing up a table with drug schedules, patient education for the inclusion of alarms on the smartphone, organization of drugs in dispensers by time and places to purchase drugs for free or at lower prices^(17)^.

With regard to dietary style, one of the main risk factors for CVD, information on cardioprotective foods published by the Ministry of Health was included, which consist of typical Brazilian foods to help patients control blood pressure, body weight, glycemic and lipid levels as well as information on the importance of controlling sodium, sugar and oil in food preparation^(18-19)^.

The practice of physical activity is one of the main actions performed for CVD prevention and control. The website brought information about the importance of aerobic physical activity and how to practice it safely, considering that the practice of physical activity reduces the risk of acute myocardial infarction reduces the risk of global mortality and all-cause mortality from all hospitalizations, with a significant reduction in hospital costs and improvement in quality of life, according to a systematic review^(5,20-21)^.

In the domain on smoking and on the use of alcohol, the main harmful effects of smoking on the cardiovascular system were presented, mainly in relation to changes in vascular endothelium, increased lipid profile, altered blood pressure levels and effects of smoking in relation to the cardiodepressant effect of alcohol^(22-23)^. In this domain, the website provided information on types of treatment for nicotine dependence, such as nicotine replacement therapies and types of drug treatment as well as places to seek help for smoking and drinking treatment^(22-23)^.

The stress and sleep domain provides information about how stress and sleep deprivation affect cardiovascular health and how performing integrative practices help in its balance. The objective of this domain was to demonstrate the benefits that sleep brings to cardiovascular health and how it prevents cases of acute myocardial infarction, because sleep is essential for the nocturnal descent of blood pressure around 10%, which favors cardiovascular protection^(5,24)^. In this domain, we brought non-drug practices that could reduce patients’ stress level. However, those that are not commonly used by the Brazilian population were excluded to avoid excess information.

The last domain portrays sexual activity, its importance for cardiovascular health and how to perform it safely, especially in patients with CVD, considering that most patients say they have a decrease in weekly frequency, a reduction in the quality of sexual activity and an increase in fear of having a new acute coronary event^(5,25)^. A study showed that of 96 patients admitted for coronary heart disease, only 4% received information from health professionals about the practice of sexual activity, demonstrating once again the importance of this topic^(26-27)^.

After building the website, its content validity evidence was verified through assessment by professional and lay experts, who verified the degree to which the instructional material focused on the purpose for which it was built. More specifically, it was assessed whether all the necessary content for guiding patients with CAD was contained on the website and whether this information was being conveyed clearly and coherently, given the current scientific evidence on the subject.

All domains were assessed by experts using a Likert scale, having been approved with a CVR equal to or greater than the critical value established for the number of judges. This study chose to perform the CVR calculation because it is considered a more critical agreement calculation with a corresponding significance calculation, which reduces the risk of overestimation in the agreement analysis. Moreover, the CVI was calculated, as recommended in a publication on content validity methods^(11-12)^.

Similar studies that developed and analyzed website validity evidence can be identified in the literature. In the study to develop an educational website for teaching the nursing process in cardiology, the methodology used to create the website was similar to that of the present study. However, website assessment was carried out by the creators themselves, with the aim of observing structural performance, not content validity^(28)^.

In a different approach^(29)^, the study of development and assessment of a website about Alzheimer’s Disease and its consequences for communication brings a content assessment carried out by 56 individuals, being 16 elderly, 12 elderly caregivers and 28 speech therapists, who judged the website assessment and content assessment. In this study, three website assessment instruments were used: one was used to characterize the study population and Internet use; the other was based on a questionnaire to assess a speech therapy and pediatrics blog, in order to assess the content about Alzheimer’s disease; and the last one, an instrument for assessing the website technical quality, was developed through a questionnaire that addresses topics related to content, accuracy, authors, updates, audience, navigation, links and structure^(29)^. This assessment was very similar to that used in the present study.

In another website design study, a website was developed to educate musicians on how to take better care of their hearing health. The site elaboration followed the same principles of the present study; however, the website content assessment was not systematized and the authors assessed musicians’ knowledge before and after reading the website content and being instructed^(30)^.

These comparisons reinforce the relevance of a complete assessment of a website in relation to content quality, usability and ease of use by the target population as well as its effectiveness in health education.

### Study limitations

Although an adequate level of validity evidence was achieved both in the assessment of professional and lay experts, this study did not assess the understanding of lay experts regarding the topic addressed. Further studies that assess the understanding of the issues covered on this website and what effect on lifestyle changes will be needed.

### Contributions to nursing, health, and public policies

The website, developed and validated in terms of content, can be useful as an auxiliary tool for health education for patients with CAD and their relatives, aiming at a better understanding of the disease and treatment by this population, in addition to the importance of incorporating a healthy lifestyle.

## CONCLUSIONS

The website for guidance of patients with CAD was developed based on national and international recommendations regarding the adoption of a healthy lifestyle, adoption of health management behavior and drug compliance measures. This website used various types of instructional resources for conveying information, such as videos, booklets, banners and interviews. Adequate content validity evidence was obtained after website assessment by professional and lay experts.
